# Identification of a novel gene pairs signature in the prognosis of gastric cancer

**DOI:** 10.1002/cam4.1303

**Published:** 2017-12-28

**Authors:** Pai‐Lan Peng, Xiang‐Yu Zhou, Guo‐Dong Yi, Peng‐Fei Chen, Fan Wang, Wei‐Guo Dong

**Affiliations:** ^1^ Department of Gastroenterology Renmin Hospital of Wuhan University Wuhan 430060 China; ^2^ Department of Gastroenterology The Central Hospital of Enshi Autonomous Prefecture Enshi 445000 China; ^3^ Department of Gastroenterology Zhongnan Hospital of Wuhan University Wuhan 430071 China

**Keywords:** Gastric cancer, gene pairs, prognosis, signature

## Abstract

Current prognostic signatures need to be improved in identifying high‐risk patients of gastric cancer (GC). Thus, we aimed to develop a reliable prognostic signature that could assess the prognosis risk in GC patients. Two microarray datasets of GSE662254 (*n* = 300, training set) and GSE15459 (*n* = 192, test set) were included into analysis. Prognostic genes were screened to construct prognosis‐related gene pairs (PRGPs). Then, a penalized Cox proportional hazards regression model identified seven PRGPs, which constructed a prognostic signature and divided patients into high‐ and low‐risk groups according to the signature score. High‐risk patients showed a poorer prognosis than low‐risk patients in both the training set (hazard ratios [HR]: 6.086, 95% confidence interval [CI]: 4.341–8.533) and test set (1.773 [1.107–2.840]). The PRGPs signature also achieved a higher predictive accuracy (concordance index [C‐index]: 0.872, 95% CI: 0.846–0.897) than two existing molecular signatures (0.706 [0.667–0.744] for a 11‐gene signature and 0.684 [0.642–0.726] for a 24‐lncRNA signature) and TNM stage (0.764 [0.715–0.814]). In conclusion, our study identified a novel gene pairs signature in the prognosis of GC.

## Introduction

Gastric cancer (GC) is one of the most common cancers around the world, with an estimated 951,600 cases and 723,100 deaths per year 1. In spite of great improvements in chemo‐, radio‐, and surgical treatment, the 5‐year overall survival rate remains unsatisfactory. This is mainly caused by advanced stages at diagnosis and high recurrence rates after treatment. Currently, TNM (tumor/node/metastasis) staging system has been widely used for prognostic prediction. However, some patients with the same TNM stage and treatment might have various clinical outcomes. Thus, it is necessary to identify the subset of patients at high risk for recurrence and death, and provide timely intervention.

The availability of large‐scale gene expression profiles brings the chance to identify more reliable prognostic signatures in various cancers. Several studies have proposed gene‐expression prognostic signatures in GC 2, 3, 4, 5, 6. However, the models based on gene expression levels of one dataset were difficult to apply in another dataset directly, considering batch effects 7. Instead, the methods based on relative ranking of gene expression levels can be used without the need for eliminating batch effects 8, 9. In this study, we constructed prognosis‐related gene pairs (PRGCs) to develop and validate a novel prognostic signature for GC.

## Method

### Data collection

Normalized gene expression profiles of GC were downloaded from Gene Expression Omnibus (GEO) database (http://www.ncbi.nlm.nih.gov/geo/). Microarray datasets were selected if fulfilling the following criteria: (1) based on the chip platform of Affymetrix Human Genome U133 Plus 2.0 Array (GPL570); (2) availability of related clinical data, especially follow‐up time and survival status; (3) sample size of more than 150. Finally, two datasets (GSE62254 and GSE15459) were included in this study. GSE62254 (*n* = 300) was used as a training set for signature identification, while GSE15459 (*n* = 192) was used as a test set for signature validation.

### Data preprocessing

The probe IDs were matched to gene symbols using the Affymetrix annotation file (http://www.affymetrix.com). When multiple probes matched to an identical gene symbol, we selected the probe ID with the largest inter‐quartile range (IQR) of expression values among all multiple probe IDs to represent the gene, which was biologically more reasonable and robust than the average method 10.

### Prognostic genes screening

Prior to analysis, we calculated mean intensities of each gene across all samples, and filtered out the un‐expressed genes (the smallest 20% rank sum of mean intensity) and un‐informative genes (the lowest 20% rank sum of standard deviations) to decrease the false discoveries. Then, survival analysis was conducted in the training set to screen prognostic genes in GC, using the log‐rank test and permutation method (*n* = 300).

### Construction of a prognostic signature based on PRGPs

The expression level of prognostic genes in a specific sample underwent pair‐wise comparison to generate a score for each PRGP. If PRG 1 was more than PRG2, a PRGP score of 1 was assigned; otherwise, the PRGP score was 0. The PRGPs score profile was used to build the prognostic signature. To minimize the risk of over‐fitting, we used a Cox proportional hazards regression model combined with the least absolute shrinkage and selection criteria operator (glmnet, version 2.0‐10) 11. The penalty parameter was estimated by 10‐fold cross‐validation at 1SE beyond the minimum partial likelihood deviance.

### Validation and evaluation of the PRGPs signature

The patients were divided into high‐ and low‐risk groups according to the PRGPs score cutoff, which was determined by a time‐dependent receiver operating characteristic (ROC) curve (survivalROC, version 1.0.3) at 5 years 12. We adopted the nearest neighbor estimation (NNE) method to estimate the ROC curve. The score corresponding to the shortest distance between the ROC curve and the point of 100% true positive and 0% false positive was used as the cutoff value. Survival differences between the high‐ and low‐risk groups were assessed by the Kaplan–Meier estimate and compared using the log‐rank test. To validate the signature, we calculated the PRGPs score profile in the test set, classified the patients into high‐ and low‐risk groups using the same cutoff value. We also compared the prognostic accuracy of PRGPs with two existing molecular signatures in terms of time‐dependent area under ROC curve (AUC) and concordance index (C‐index) (survcomp, version 1.22.0 and compareC, version 1.3.1) 13.

### Gene set enrichment analysis

To identify potential biological processes related with the risk based on the PRGPs signature, Gene set enrichment analysis (GSEA) (http://software.broadinstitute.org/gsea/index.jsp) was conducted to detect whether a series of priori defined biological processes were enriched in the gene rank derived from differentially expressed genes (DEGs) between the high‐ and low‐risk groups. False discovery rate (FDR) <0.05 was chosen as the cut‐off criteria.

### Statistical analysis

All statistical analyses were performed using R (version 3.3.1, https://www.r-project.org/). For use with GSEA software, the collection of annotated gene sets of h.all.v5.2.symbols.gmt in Molecular Signatures Database (MSigDB, http://software.broadinstitute.org/gsea/msigdb/index.jsp) was chosen as the reference gene sets. A two‐sided *P* value <0.05 was considered statistically significant.

## Results

### Construction and definition of the PRGPs signature

**Figure 1:**
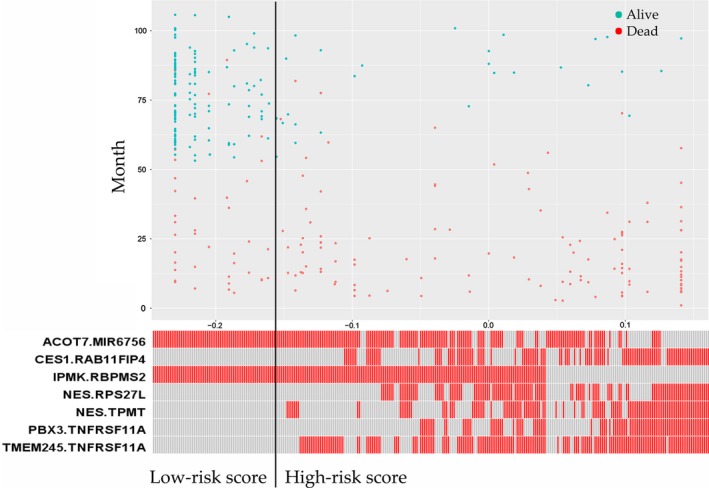
Distribution of the patients’ survival status and risk scores of the prognosis‐related gene pairs (PRGPs) signature.

**Figure 2:**
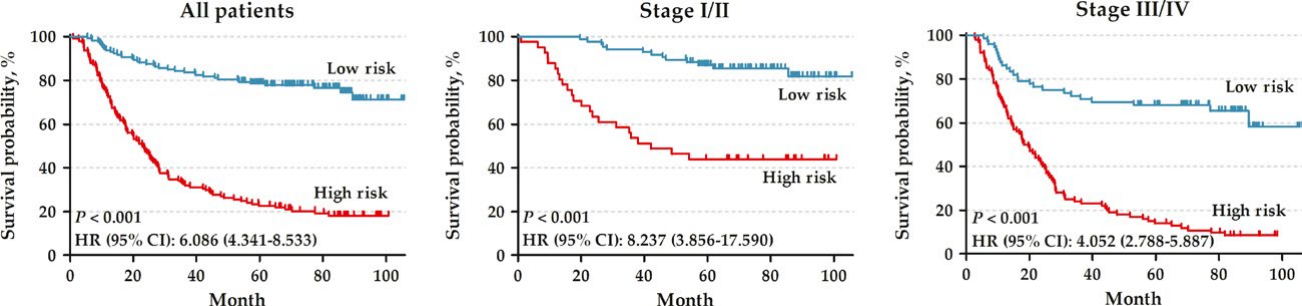
Kaplan–Meier curves of overall survival among the patients in training set.

In the training set, there were a total of 300 GC patients (199 male [66%] and 101 female [34%]; median age [range]: 64 [24–86] years). A total of 518 prognostic genes were identified to construct 133903 PRGPs. Then, we constructed a risk score consisting of seven PRGPs using L1‐penalized Cox proportional hazards regression (Fig. 1). The PRGPs signature consisted of 12 unique prognostic genes (ACOT7, CES1, IPMK, NES, PBX3, TMEM245, MIR6756, RAB11FIP4, RBPMS2, RPS27L, TPMT, and TNFRSF11A) (Table 1). In time‐dependent ROC curve analysis, the optimal cutoff for the signature to classify patients into high‐ and low‐risk groups was set at −0.154. High‐risk patients showed a poorer prognosis than low‐risk patients (hazard ratios [HR]: 6.086, 95% confidence interval [CI]: 4.341–8.533) (Fig. 2). Subgroup analysis showed consistent results (Table**** 2).

**Table 1:** Signature information

Gene pair 1	Full name	Gene pair 2	Full name	Coefficient
ACOT7	Acyl‐CoA thioesterase 7	MIR6756	microRNA 6756	−0.038007046
CES1	Carboxylesterase 1	RAB11FIP4	RAB11 family interacting protein 4	0.024780097
IPMK	Inositol polyphosphate multikinase	RBPMS2	RNA‐binding protein with multiple splicing 2	−0.190466701
NES	Nestin	RPS27L	Ribosomal protein S27‐like	0.043310157
NES	Nestin	TPMT	Thiopurine S‐methyltransferase	0.010844818
PBX3	Pre‐B‐cell leukemia homeobox 3	TNFRSF11A	TNF receptor superfamily member 11a	0.048826825
TMEM245	Transmembrane protein 245	TNFRSF11A	TNF receptor superfamily member 11a	0.014605079

**Table 2:** Subgroup analysis for the hazard ratios (HRs) between high‐ and low‐risk groups divided by the prognosis‐related gene pairs (PRGPs) signature

Variables	No. of patients		HR (95% CI)	Log‐rank *P* value
	High risk	Low risk		
All	141	159	6.086 (4.341–8.533)	<0.0001
Age				
<65 years	78	83	6.583 (4.049–10.700)	<0.0001
>65 years	63	76	6.251 (3.881–10.070)	<0.0001
Sex				
Male	93	106	5.830 (3.846–8.838)	<0.0001
Female	48	53	6.321 (3.552–11.250)	<0.0001
Lauren type				
Intestinal	53	93	8.423 (4.713–15.050)	<0.0001
Diffuse	75	59	4.671 (2.965–7.359)	<0.0001
Molecular subtype				
MSS/TP53−	60	47	5.620 (3.316–9.524)	<0.0001
MSS/TP53+	28	51	7.531 (3.599–15.760)	<0.0001
Differentiation				
Well/moderate	38	76	12.250 (6.150–24.410)	<0.0001
Poor	55	61	6.172 (3.620–10.520)	<0.0001
TNM stage				
I/II	41	85	8.237 (3.856–17.590)	<0.0001
III/IV	100	72	4.052 (2.788–5.887)	<0.0001
Lymphovascular invasion				
Positive	103	102	4.558 (3.107–6.686)	<0.0001
Negative	25	48	25.440 (9.711–66.650)	<0.0001
Venous invasion				
Positive	25	19	9.201 (4.055–20.880)	<0.0001
Negative	47	82	10.170 (5.474–18.900)	<0.0001
Perineural invasion				
Positive	51	37	4.751 (2.799–8.064)	<0.0001
Negative	60	99	10.050 (5.730–17.640)	<0.0001

HR, hazard ratio; CI, confidence interval.

Moreover, we also conducted a sensitivity analysis by omitting one gene pair per time to evaluate the effect of each included gene pair on the signature, and found that each included gene pair helped improve the predictive ability of the signature (Table S1).

### Validation of the PRGPs signature

**Figure 3:**
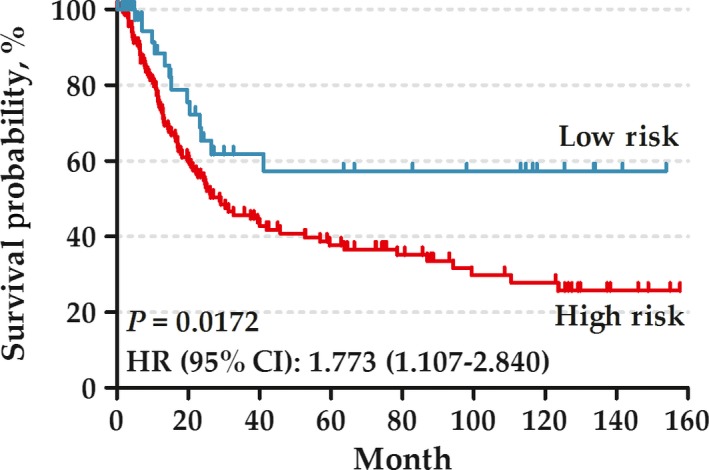
Kaplan–Meier curves of overall survival among the patients in test set.

In the test set, there were a total of 192 GC patients (125 male [65%] and 67 female [35%]; median age [range]: 67 [23–92] years). According to the same signature score and cutoff value with the training set, high‐risk patients also showed a poorer prognosis than low‐risk patients in the test set (HR: 1.773, 95% CI: 1.107–2.840) (Fig. 3).

Furthermore, we randomly selected 14 genes from the 518 prognostic genes to construct seven gene pairs as pseudo‐PRGPs. The pseudo‐PRGPs signature showed a poor predictive accuracy in the prognosis of GC (AUC at 5 years = 0.620), which proved the methodological reliability in this study (Fig. S1).

### Comparison with other prognostic signatures

**Figure 4:**
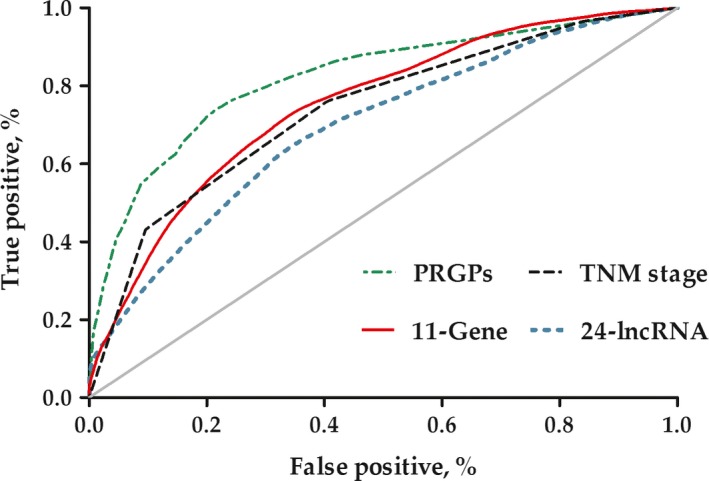
Time‐dependent receiver operating characteristic (ROC) curves of different prognostic signatures in gastric cancer.

We also compared the PRGPs signature with two existing molecular signatures, both of which were also generated from GSE62254. The 24‐lncRNA expression signature was reported with an AUC of 0.71, and 0.769 for the 11‐Gene expression signature [3,4]. After modeling again in the training set, both the signatures showed a lower predictive efficiency than the PRGPs signature (AUC at 5 years = 0.872, 0.751 and 0.695 for PRGPs, 11‐Gene and 24‐lncRNA), as well as for TNM stage (AUC at 5 years = 0.737) (Fig. 4). Furthermore, the GRGPs signature achieved a higher predictive accuracy than the 11‐Gene signature (C‐index [95% CI]: 0.872 [0.846–0.897] vs. 0.706 [0.667–0.744]; *P *=* *0.026) and the 24‐lncRNA signature (C‐index [95% CI]: 0.872 [0.846–0.897] vs. 0.684 [0.642–0.726]; *P *=* *0.011), and moderately higher than the TNM stage signature (C‐index [95% CI]: 0.872 [0.846–0.897] vs. 0.764 [0.715–0.814]; *P *=* *0.077) (Table 3).

**Table 3:** Predictive accuracy of different prognostic signatures in gastric cancer

Signature	AUC	C‐index (95% CI)	*P* value^a^
PRGPs	0.820	0.872 (0.846–0.897)	–
TNM stage	0.737	0.764 (0.715–0.814)	0.077
11‐Gene	0.751	0.706 (0.667–0.744)	0.026
24–lncRNA	0.695	0.684 (0.642–0.726)	0.011

AUC, area under time‐dependent receiver operating characteristics (ROC) curve; CI, confidence interval; PRGPs, prognosis‐related gene pairs; C‐index, concordance index.

a Represents the difference between the PRGPs and other signatures in terms of C‐index.

### Biological processes associated with the PRGPs signature

**Figure 5:**
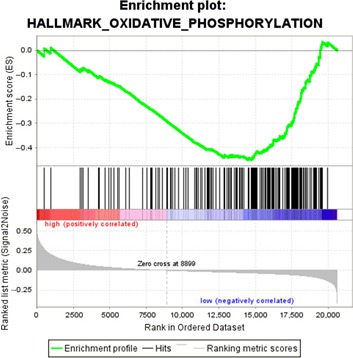
Gene set enrichment analysis.

After the patients were divided into high‐ and low‐risk groups according to the PRGPs signature, GSEA identified one gene set of “oxidative and phosphorylation” significantly enriched in the low‐risk group (FDR = 0.029) (Fig. 5). The set contained 188 genes, among which there exited a complex protein–protein interaction network (Fig. S2). When setting the selection criteria at FDR < 0.1, five more enriched sets were identified, namely “MYC targets,” “interferon *α* response” and “E2F targets” in the low‐risk group and “myogenesis” in the high‐risk group.

## Discussion

With the development of high‐throughput gene detection technology, we were entering a new era of big biological data. A tremendous amount of genomic information was detected in individual samples, which promoted the identification of novel diagnostic, prognostic, predictive or pharmacodynamic biomarkers 14. Effective development and validation of biomarkers depended mainly on the intended use. A genomic signature was a biomarker in which the genomic data were combined in a defined manner to provide either a continuous score or a categorical classifier for clinical decision‐making.

Prognostic signatures were baseline measurement to provide information about the long‐term outcome for cancer patients. Currently, the microarray or RNA‐sequencing data of gene mutation and expression were usually used to construct novel prognostic signatures by a Cox proportional hazards regression model 15, 16. Well‐developed and validated prognostic signatures could help improve patient management in a personalized manner. However, most of these signatures has not been accepted or widely used in clinical practice. This was caused by multiple factors. First of all, the method based on gene expression was difficult to integrate the samples in different sets for batch effects, which limited the sample size. Second, all signatures showed a significant association with the prognosis, but most failed to provide a specific risk‐score formula and the cutoff value for high‐ and low‐risk groups.

In this study, we constructed a risk score consisting of seven GRGPs using L1‐penalized Cox proportional hazards regression. The calculated score by this gene pair‐based method was based entirely on the gene expression profile of one GC patient, and could be used without the need for eliminating batch effects. Thus, the formula and cutoff value could be used across multiple datasets, which was an important advantage. When taking the same formula and cutoff value in the test set, we also reached a consistent result. This indicted the robustness of the method and the PRGPs signature. Accordingly, the PRGPs signature showed a higher predictive efficiency and accuracy than other prognostic signatures.

The seven GRGPs consisted of 12 genes, among which two genes (CES1 and TNFRSF11A) were also part of the 11‐Gene signature. Of these 12 genes, only PBX1 has been investigated for potential mechanism in GC 17, 18. We thought the other 11 genes might also play a role in GC. Moreover, the expression imbalance in certain gene pairs might play a more important role than individual differentially expressed genes. In GSEA, we found that “oxidative and phosphorylation” was significantly enriched in the low‐risk group, which was consistent with the recent study 19.

The limitations should be acknowledged for our study. First, this was a retrospective designed study, rather than a prospective cohort study. Second, the sample size was relatively small, although the method we developed could eliminate the batch effects. Third, we only considered the microarray data based on GLP570 and ignored other unusual platforms, which might lead to selection bias.

In conclusion, our study identified a novel gene pairs signature in the prognosis of GC.

## Conflict of Interest

None.

## Supporting information


**Table S1.** Sensitivity analysis for the prognosis‐related gene pairs signature.
**Figure S1.** Time‐dependent receiver operating characteristic (ROC) curveof the pseudo prognosis‐related gene pairs signature.
**Figure S2.** Protein–protein interaction network of genes inthe enriched set of “oxidationand phosphorylation”.
