# Supplementary material for: Identification of a novel gene pairs signature in the prognosis of gastric cancer
Source: Cancer Med. 2017 Dec 28;7(2):344–50. doi: 10.1002/cam4.1303 (PMC5806102; doi:10.1002/cam4.1303)
Supplement: Supplementary file 1 — Table S1. Sensitivity analysis for the prognosis‐related gene pairs signature. Figure S1. Time‐dependent receiver operating characteristic (ROC) curveof the pseudo prognosis‐related gene pairs signature. Figure S2. Protein–protein interaction network of genes inthe enriched set of “oxidationand phosphorylation”. [file CAM4-7-344-s001.pdf]

Table S1. Sensitivity analysis for the prognosis-related gene pairs signature.

Gene pair omitted	AUC
ACOT7-MIR6756	0.806
CES1-RAB11FIP4	0.813
IPMK-RBPMS2	0.806
NES-RPS27L	0.798
NES-TPMT	0.816
PBX3-TNFRSF11A	0.802
TMEM245-TNFRSF11A	0.808
Overall	0.820

AUC, area under time-dependent receiver operating characteristics (ROC) curve.

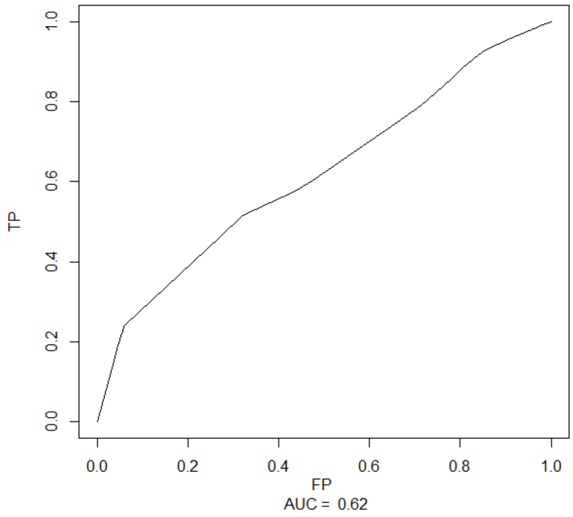

Figure S1. Time-dependent receiver operating characteristic (ROC) curve of the pseudo prognosis-related gene pairs signature.

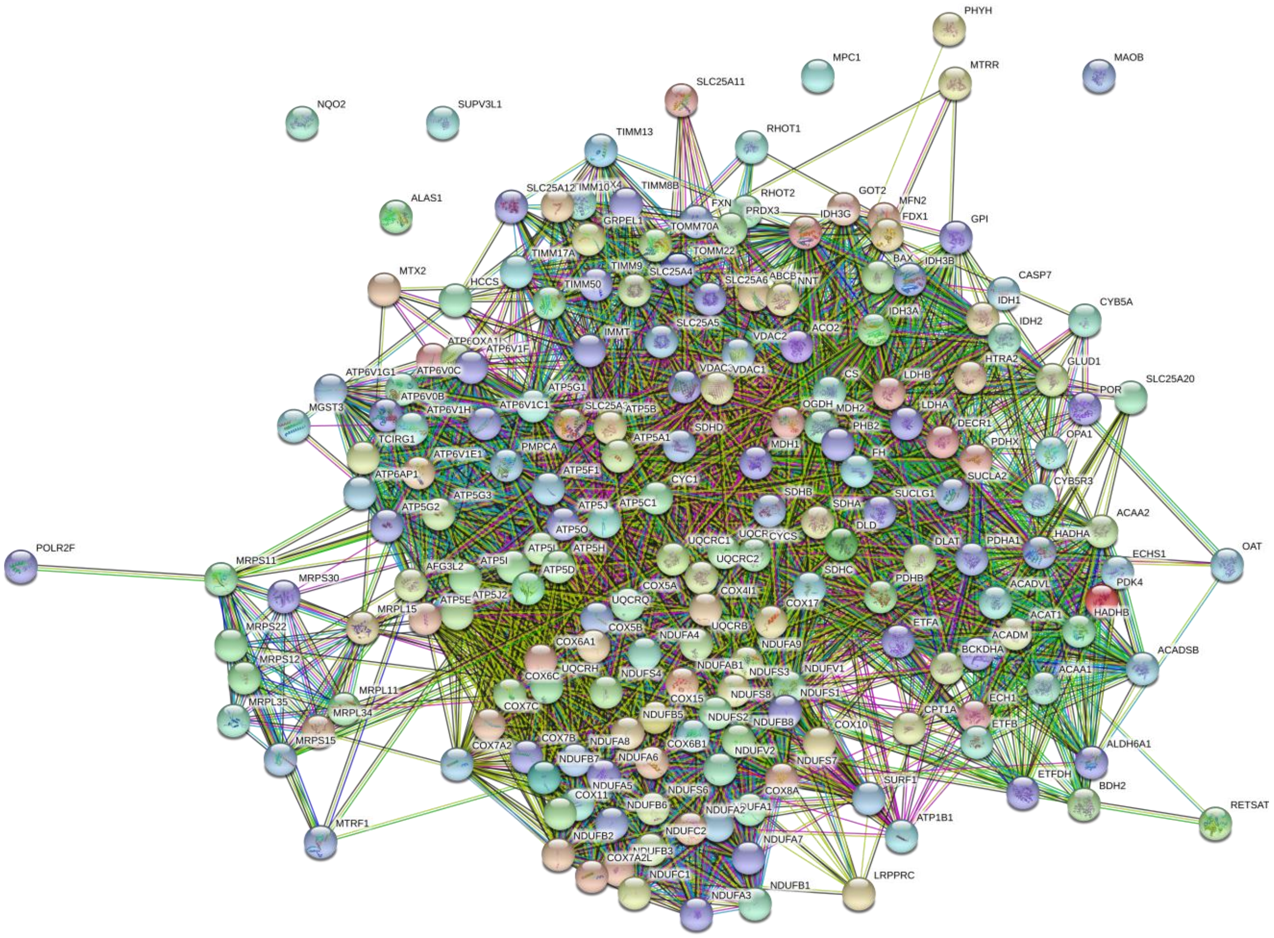

Figure S2. Protein–protein interaction network of genes in the enriched set of “oxidation and phosphorylation”.
